# MEANINGFUL ACTIVITY ENGAGEMENT AND WELL-BEING AMONG DISABLED OLDER ADULTS: THE MODERATED ROLE OF ENVIRONMENT

**DOI:** 10.1093/geroni/igac059.2003

**Published:** 2022-12-20

**Authors:** Yi-Hsuan Tung, Iris Chi, Shinyi Wu, Shiau-Fang Chao

**Affiliations:** University of Southern California, Los Angeles, California, United States; University of Southern California, Los Angeles, California, United States; University of Southern California, Los Angeles, California, United States; National Taiwan University, Taipei, Taipei, Taiwan (Republic of China)

## Abstract

Engaging in meaningful activities has been seen as an important way to sustain the well-being of older adults with disabilities and to achieve person-centered care. Yet, it is still unclear whether and to what extent meaningful activity engagement promotes well-being for community-dwelling older adults with disabilities, and how the environmental factors could affect these relationships. This study aims to investigate the relationship between meaningful activity engagement and psychological well-being, and to explore the moderated role of environmental factors (physical, attitudinal, service/support, policy). Survey data conducted in Taiwan between April and July of 2018 were analyzed by using multiple regression (N=1,244). Three types of meaningful activities (instrumental, social, and leisure) were identified based on a self-rated activity meaningfulness measure. Findings showed that higher levels of engagement in three types of meaningful activities were associated with better quality of life (QOL), but only engaging in meaningful leisure was associated with less depressive symptoms while adjusting functional status. Perceived better policy-related environment (e.g., long-term care services) could reinforce the positive effects of three types of meaningful activity engagement on QOL and depressive symptoms. Findings also indicated perceived attitudinal environment moderated the association between meaningful leisure activity engagement and QOL. These results established the influences of meaningful activity engagement on the well-being of community-dwelling older adults with disabilities and highlight the importance of age-friendly environment in supporting meaningful activity engagement and older adults’ well-being.

